# Brief Research Report: Anti-SARS-CoV-2 Immunity in Long Lasting Responders to Cancer Immunotherapy Through mRNA-Based COVID-19 Vaccination

**DOI:** 10.3389/fimmu.2022.908108

**Published:** 2022-07-05

**Authors:** Marta Sisteré-Oró, Diana D. J. Wortmann, Naína Andrade, Andres Aguilar, Clara Mayo de las Casas, Florencia Garcia Casabal, Susana Torres, Eduardo Bona Salinas, Laura Raventos Soler, Andrea Arcas, Carlos Esparre, Beatriz Garcia, Joselyn Valarezo, Rafael Rosell, Roberto Güerri-Fernandez, Maria Gonzalez-Cao, Andreas Meyerhans

**Affiliations:** ^1^ Infection Biology Laboratory, Department of Medicine and Life Sciences, Universitat Pompeu Fabra (UPF), Barcelona, Spain; ^2^ Instituto Oncologico Dr Rosell, Hospital Quiron-Dexeus Barcelona, Barcelona, Spain; ^3^ Laboratorio Oncología, Hospital Universitario Dexeus, Pangaea Oncology Lab, Barcelona, Spain; ^4^ Laboratorio Oncología, Germans Trias i Pujol Health Science Insitute and Hospital (IGTP), Badalona, Spain; ^5^ Infectious Diseases Unit, Hospital del Mar, Universitat Autònoma de Barcelona, Barcelona, Spain; ^6^ Catalan Institution for Research and Advanced Studies (ICREA) Pg. Lluiís Companys 23, Barcelona, Spain

**Keywords:** COVID-19 vaccination, mRNA-based vaccines, cancer, SARS-CoV-2, long lasting responders, immunotherapy, immune checkpoint inhibitors (ICIs)

## Abstract

Cancer patients (CPs) have been identified as particularly vulnerable to SARS-CoV-2 infection, and therefore are a priority group for receiving COVID-19 vaccination. From the patients with advanced solid tumors, about 20% respond very efficiently to immunotherapy with anti-PD1/PD-L1 antibodies and achieve long lasting cancer responses. It is unclear whether an efficient cancer-specific immune response may also correlate with an efficient response upon COVID-19 vaccination. Here, we explored the antiviral immune response to the mRNA-based COVID-19 vaccine BNT162b2 in a group of 11 long-lasting cancer immunotherapy responders. We analysed the development of SARS-CoV-2-specific IgG serum antibodies, virus neutralizing capacities and T cell responses. Control groups included patients treated with adjuvant cancer immunotherapy (IMT, cohort B), CPs not treated with immunotherapy (no-IMT, cohort C) and healthy controls (cohort A). The median ELISA IgG titers significantly increased after the prime-boost COVID vaccine regimen in all cohorts (Cohort A: pre-vaccine = 900 (100-2700), 3 weeks (w) post-boost = 24300 (2700-72900); Cohort B: pre-vaccine = 300 (100-2700), 3 w post-boost = 8100 (300-72900); Cohort C: pre-vaccine = 500 (100-2700), 3 w post-boost = 24300 (300-72900)). However, at the 3 w post-prime time-point, only the healthy control group showed a statistically significant increase in antibody levels (Cohort A = 8100 (900-8100); Cohort B = 900 (300-8100); Cohort C = 900 (300-8100)) (P < 0.05). Strikingly, while all healthy controls generated high-level antibody responses after the complete prime-boost regimen (Cohort A = 15/15 (100%), not all CPs behaved alike [Cohort B= 12/14 (84'6%); Cohort C= 5/6 (83%)]. Their responses, including those of the long-lasting immunotherapy responders, were more variable (Cohort A: 3 w post-boost (median nAb titers = 95.32 (84.09-96.93), median Spike-specific IFN-γ response = 64 (24-150); Cohort B: 3 w post-boost (median nAb titers = 85.62 (8.22-97.19), median Spike-specific IFN-γ response (28 (1-372); Cohort C: 3 w post-boost (median nAb titers = 95.87 (11.8-97.3), median Spike-specific IFN-γ response = 67 (20-84)). Two long-lasting cancer responders did not respond properly to the prime-boost vaccination and did not generate S-specific IgGs, neutralizing antibodies or virus-specific T cells, although their cancer immune control persisted for years. Thus, although mRNA-based vaccines can induce both antibody and T cell responses in CPs, the immune response to COVID vaccination is independent of the capacity to develop an efficient anti-cancer immune response to anti PD-1/PD-L1 antibodies.

## Introduction

The pandemic caused by the new severe acute respiratory syndrome coronavirus 2 (SARS-CoV-2) has a strong pathogenic impact on different subgroups of the general population (1). This generates the need to test efficacies of the rapidly developed COVID-19 vaccines in particularly vulnerable groups which have been excluded from the initial vaccine efficacy studies. Amongst these groups are immunocompromised individuals that are with active cancers, that receive immunosuppressive drugs i.e. after organ transplantation or that are infected with human immunodeficiency viruses (2–5). Due to their immunocompromised state, these individuals have a significantly higher mortality upon SARS-CoV-2 infection than non-compromised individuals and thus should be taken care of with high priority (6, 7). However, there are now CPs that generate exceptional cancer responses after immunotherapy with anti-PD-1/PD-L1 antibodies and that achieve a long survival time (8, 9). Such patients may possibly respond better to COVID-19 vaccination or may experience vaccine toxicity *via* an exaggerated immune response.

The mRNA-based COVID-19 vaccines from BioNTech/Pfizer and Moderna have been developed in record-speed and were given emergency approval in December 2020 after demonstrating to be safe and highly efficient (10, 11). They are inducing virus-specific antibody and cellular immunity, and about a 95% protection from SARS-CoV-2-induced disease (12, 13). However, vaccine immunogenicity in immunocompromised individuals is expected to be diminished to various degrees depending on the particular condition and state of immunosuppression (2, 14–17). Here we report vaccination outcomes in a small cohort of solid CPs including a cohort of long survivors under immunotherapy (cohort B, subgroup B.1) with checkpoint inhibitors (mainly anti PD-1/PD-L1 antibodies) and relate them to COVID-19 vaccine studies of cancer patients that have been published until January 2022 (3, 14, 16, 18–28).

## Materials and Methods

### Sample Collection

This is a post-authorization observational study that prospectively collected data and blood samples from CPs treated with anti PD-1/PD-L1 antibodies that were vaccinated with mRNA-based COVID-19 vaccines (cohort B, subgroups B.1 and B.2). Subgroup B.1 consists of long-lasting responders, which are CPs with advanced tumors that survive longer than the expected median survival achieved with non-immunological therapies. Subgroup B.2 consists of CPs under adjuvant therapy after receiving an initial primary therapy, either radiation or surgery. Adjuvant therapy is given to help prevent cancer relapse. The study included other cohorts as controls: one of CPs not treated with anti-PD-1 antibodies (cohort C) and a second cohort of healthy individuals vaccinated against COVID-19 (cohort A). From 14 patients treated with immunotherapy (cohort B), 11 were on active treatment (cohort B.1) and had received their last dose of anti PD-1/PD-L1 antibody within a median of 31 days (range 42 days to 21 days) before COVID-19 vaccination. Three patients (cohort B.2) had discontinued anti PD-1/PD-L1 treatment 8 months, 10 months and 14 months before COVID-19 vaccination due to immune-related adverse events that were finally resolved. These three patients maintained their cancer response in spite of not being under active anticancer treatment. From 6 CPs not treated with immunotherapy (cohort C), 4 were on active anticancer treatment: three of them with daily oral therapy (one with BRAF plus MEK (tyrosin-kinase inhibitors (TKIs)) and two with hormonal therapy), and 2 patients were on treatment with chemotherapy that received their last dose 14 days and 20 days before COVID-19 vaccination, respectively.

Participants received anti-COVID vaccinations following the standard schedule. The primary endpoint was to describe the specific IgG serum antibody response, the virus neutralizing capacity of these antibodies, and the T cell response. The study analyzed blood samples pre-vaccination, post-prime, and 3 weeks (w) and 9 w after the boost. The protocol of the study was approved by the institutional review board of “Grupo Hospitalario Quirón Salud-Catalunya”. Written informed consent was obtained from each patient.

### Quantification of SARS-CoV-2 Spike-Specific IgG Antibody Responses and RBD Neutralization Capacity of Patient Sera

SARS-CoV-2-specific IgG antibodies towards the full-length SARS-CoV-2 S protein were assessed by an enzyme-linked immunosorbent assay (ELISA). Costar 96-well flat-bottom, high-binding plates (2240096, Bio-Rad) were coated with 2 μg/mL full-length SARS-CoV-2 S protein (40592-V08B1, Sino Biologicals) and blocked with 3% BSA (A4503, Sigma-Aldrich) in 1x PBS. Three-fold serial dilutions of serum samples were added in duplicates to the corresponding wells. Next, a detection step using an IgG HRP antibody (A18811, Life technologies) was performed and 3,3’,5,5’-Tetramethylbenzidine (TMB) substrate (T0440-100mL, Sigma-Aldrich) was dispensed and stopped with 1N H_2_SO_4_. Finally, absorbance was measured at 450 nm using a microplate reader (iMark™ Microplate Absorbance Reader #1681130, Bio-Rad). For each sample dilution duplicate, the mean absorbance was calculated. Endpoint-titres of S-specific binding antibodies were defined as the reciprocal of the last serum dilution that provided 3 times the mean optical density of the negative control (wells without sera). Endpoint-titres above a dilution of 1:2100 were scored positive.

Neutralising antibodies against the receptor binding domain (RBD) of the S protein were evaluated from patient sera using a commercial NeutraLISA assay (EI 2606-9601-4, Euroimmune). Sera were always analysed in duplicates and processed according to the manufacturer’s instructions (EI 2606-9601-4, Euroimmune). A microplate reader (iMark Microplate Reader, Bio-Rad) was used to measure the optical densities of the plates. Percentages of inhibition (% IH) against SARS-COV-2 were calculated as detailed by the assay’s manual: % IH = 100% - [(extinction sample x 100%)/average extinction blank]. Percentages of IH were interpreted as positive if ≥35%, as doubtful if ≥20 and <35, and as negative if <20.

### Quantification of Spike-Specific T Cells From PBMCs

SARS-CoV-2-specific T cells were determined using an IFN-γ enzyme-linked immunospot (ELISpot) assay (3420-2H, Mabtech) according to the manufacturer’s instructions. Briefly, 250,000 PBMCs per well were incubated with SARS-CoV-2 spike peptides at a final concentration of 2 μg/ml for 16 - 24h (JPT-PM-WCPV-S, Sino Biologicals). A CEF peptide pool (JPT-PM-CEF-S1, Sino Biologicals, 2 ug/mL) and PMA (P8139, Sigma, 15ng/mL) plus ionomycin (I0634, Sigma, 250ng/mL) were used as positive controls. To quantify antigen-specific responses, mean spots of the RPMI control wells were subtracted from the positive wells, and the results were expressed as spot-forming units (SFU) per 10^6^ PBMCs. T cell responses were considered positive if the results were ≥14 IFN-γ-secreting cells per 10^6^ PBMCs.

### Statistics

The sample size for the study was not based on statistical hypothesis testing. The primary objective was to evaluate by descriptive summary statistics the immunogenicity at different times points in three cohorts of patients, including one cohort of long survivors treated with anti PD-1/PD-L1 antibodies. Statistical analyses and graphical representations of all data were performed using GraphPad PRISM7. For comparison of means and P-value determinations, the Mann-Whitney-U and the Kruskal Wallis test were conducted. P-values < 0.05 were considered significant.

## Results

Thirty-five persons participated in the study, including 20 CPs. Fourteen patients were on treatment with anti PD-1/PD-L1-based immunotherapy: three of them in the adjuvant setting (cohort B, subgroup B.2) and eleven patients were long lasting responders to immune-based therapies in advanced stage of cancer disease (cohort B, subgroup B.1). The study included two control cohorts: 6 CPs not treated with anti-PD-1/PD-1-L antibodies (cohort C) and 15 healthy persons (cohort A). All persons received after inclusion two doses of the BNT162b2 vaccine (prime at day 0 and boost after 21 days), except two persons from the healthy cohort that received ChAdOx1-S (boost after 90 days) and Ad26.COV2.S (one single dose), respectively. No previous infection by SARS-CoV-2 was recorded at inclusion in the study.

Noteworthy, all patients included in the immunotherapy cohort, were patients with a good control of the cancer disease, including 11 patients with long lasting responses to immunotherapy (six in complete response and five in partial response; median time on treatment was 36 months -range 12 months to 72 months-) (cohort B, subgroup B.1) and three patients with no active cancer disease and treated with immunotherapy in the adjuvant setting with anti-PD-1 antibodies (two as single agent and one patient in combination with bempegaldesleukin) (cohort B, subgroup B.2). From the cohort of CPs with non-immunologic treatments (cohort C), only one patient was in cancer progression at the time of being included into the current study. In cohort B, cancer treatment consisted of single anti-PD-1 or anti-PD-L1 antibodies in seven (63%) and three (27%) cases, respectively, and one (9%) patient received chemotherapy in combination with an anti-PD-1 antibody. From cohort C, two (33%) patients were under treatment with chemotherapy, one (17%) patient with targeted drugs against BRAF mutations and two (33%) patients were on treatment with hormonotherapy. Additional patient characteristics are included in **Table 1**. There was no apparent increase in adverse events due to the BNT162b2 vaccine, as well as no increase in immune related adverse events due to cancer immunotherapy.

**Table 1:** Patient characteristics.

	Cohort AHealthy persons(n=15)	Cohort BCancer patients IMT treatment (n=14)	Cohort CCancer patients no-IMT treatment (n=6)
**Characteristics**			
**Age – y, Median (range)**	53 (26-72)	67 (38-84)	59 (47-61)
**Sex – n (%)**			
Female	8 (53)	7 (50)	4 (67)
**ECOG PS – n (%)**			
0	15 (100)	7 (50)	4 (67)
1	0	7 (50)	2 (33)
**Cancer type– n (%)**			
Non-squamous NSCLC	NA	7 (50)	2 (33)
Squamous NSCLC	NA	3 (21)	0
Melanoma	NA	3 (21)	2 (33)
Colon cancer	NA	1 (6)	0
Breast cancer	NA		2 (33)
**Tumor stage – n (%)**			
II	NA	1 (7)	1 (17)
III	NA	3 (21)	0
IV	NA	10 (71)	5 (83)
**Cancer control – n (%)**			
No active disease	NA	3 (21)	1 (17)
Complete response	NA	6 (43)	2 (33)
Partial response	NA	5 (36)	2 (33)
Progression disease	NA	0	1 (17)
**Anticancer drug– n (%)**			
Nivolumab	0	2 (14)	0
Pembrolizumab	0	6 (43)	0
Durvalumab	0	1 (7)	0
Atezolizumab	0	3 (21)	0
Pembrolizumab-ChT	0	1 (7)	0
Nivolumab-bempegaldesleukin	0	1 (7)	0
Cht	0	0	2 (33)
BRAFi-MEKi	0	0	1 (17)
Hormonotherapy	0	0	2 (33)
No anticancer treatment	0	0	1 (17)

NA, Not applicable.

**Figure 1:**
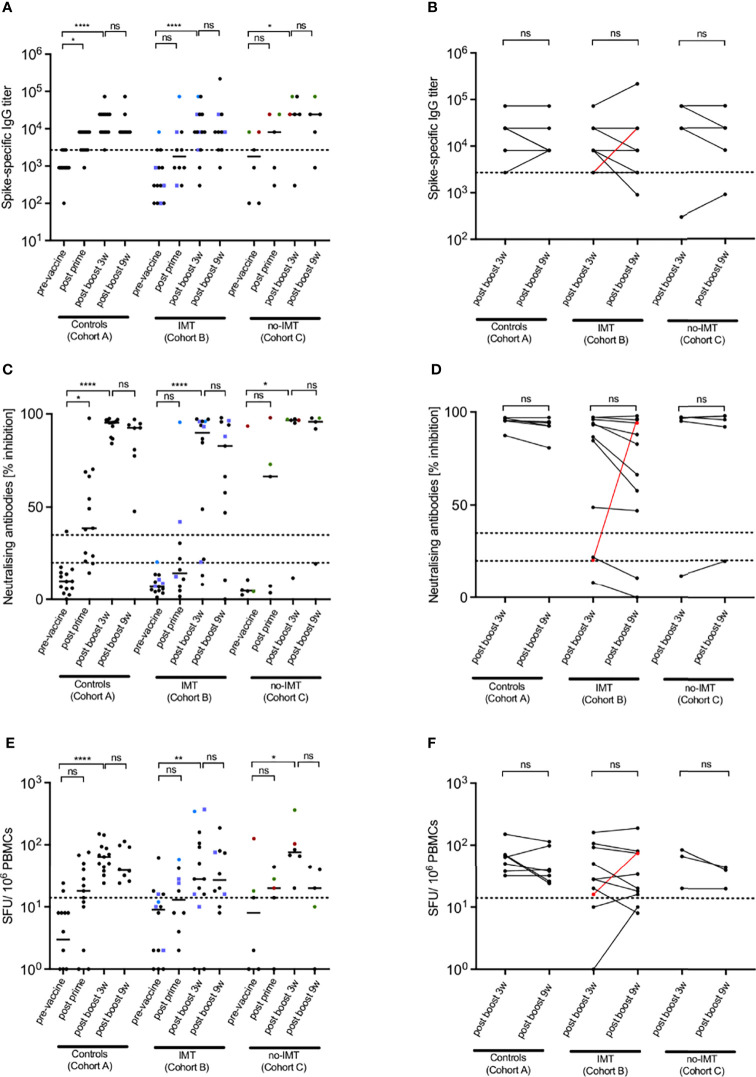
SARS-CoV-2-specific immune responses after COVID-19 vaccination. Spike-specific IgG titers **(A, B)**, nAb titers **(C, D)** and spike-specific T cell responses **(E, F)** in healthy donors (Cohort A), CP under immunotherapy (anti-PD1/PD-L1) (IMT; Cohort B, subgroups B.1 and B.2) and CP not treated with immunotherapy (no-IMT; Cohort C) after two doses of mRNA-based COVID-19 vaccines. Individuals from subgroups B.1 are represented by circles and individuals from subgroup B.2 are represented by blue squares. Dotted lines indicate positive thresholds for Spike-specific IgG and T cells. In case of nAbs **(C, D)**, the dotted line determines the thresholds for negative, intermediate, and positive results according to manufacturer’s instructions. Patients with a supposedly previous asymptomatic SARS-CoV-2 infection are differently coloured (red circled symbols for CP19; blue circled symbols for CP7 and green circled symbols for CP16) and excluded from statistical analyses. Bars represent medians. Differences between the groups were calculated using Mann– Whitney test or Kruskal-Wallis test for comparison of two or three groups. Non-significant differences were indicated as “ns”. P-values below 0.05 were considered significant and wereindicated by asterisks: *p < 0.05; **p < 0.01; ***p < 0.001; ****p < 0.0001.

To assess SARS-CoV-2-specific humoral immune responses induced by the mRNA-based COVID-19 vaccine from Pfizer/BioNTech, ELISAs against the Spike protein (S) of the SARS-CoV-2 Wuhan isolate were carried out at 4 defined time-points, pre-vaccine, 3 w post prime, 3 w post boost and 9 w post boost. The median ELISA IgG titres significantly increased after the prime-boost vaccine regimen in all cohorts (**Figure 1A**) demonstrating that CPs were responsive to the vaccine and generated virus-specific antibodies. However, at the 3 w post-prime time-point, only cohort A (healthy control group) showed a statistically significant increase in antibody levels (P < 0.05). The respective increase in cohorts B and C was more variable and did not reach significance. Importantly, not all of the CPs generated high level antibody responses after the complete prime-boost regimen (Cohort B, 12/14=85,7%; Cohort C, 5/6 = 83%), while all individuals of the cohort A of healthy controls did (15/15=100%). Together this supports the strong immunogenicity of mRNA-based COVID-19 vaccines and reveals the immunocompromised state of some CPs, including also 2 cases from the cohort B of long lasting cancer immune responders.

To further access vaccine-induced neutralising antibodies and virus-specific cellular immune responses, NeutraLISA and IFN-γ ELISpot assays were performed. The results were comparable to those of the ELISA assays. All cohorts experienced a significant increase in neutralising antibodies (**Figure 1C**) and cellular Th1 responses (**Figure 1E**) demonstrating the capacity of the mRNA-based vaccines to induce both antibody and T cell responses. Again, the response was more variable in cohorts B (B.1 and B.2) and C than in the healthy controls from cohort A, and not all cancer CPs responded to the vaccines (non-responders for the nAb: cohort B=28.6%, cohort C=17%; non-responders for the ELISPOT: cohort B=21.5%, cohort C=0%). A simple overview of all responses of the different groups is given in **Figure S1**. To better visualize differences within the subgroups of cohort B, we plotted the responses of B.1 and B.2 in **Figure S2**.

Noteworthy, there was one individual from cohort C with advanced breast cancer in complete response to hormonotherapy (CP19; red dots in **Figures 1A, C, E**) that showed virus-specific IgG and Th1 responses, and a high neutralising antibody response before starting the vaccination scheme. This patient had been probably infected by SARS-CoV-2 previously. Nevertheless, before inclusion in the study, CP19 did neither have symptoms of a SARS-COV-2 infection nor was previously diagnosed of being infected. Interestingly, while vaccination increased the specific antibody response, the respective Th1 response was diminished. Patients CP7 (blue dots in **Figures 1A, C, E**) (metastatic non-small cell lung cancer in complete response to anti-PD-L1 antibody; cohort B, subgroup B.1) and CP16 (green dots in **Figures 1A, C, E**) (metastatic melanoma in progression after several lines of treatment, cohort C) were potentially SARS-CoV-2-infected before vaccination as they had clear positive S-specific IgG titres and borderline Th1 responses. Upon their first vaccination, both developed strong neutralising antibody responses and showed an increase in their specific T cells. However, as CP7 obtained anti-PDL1 antibodies just 1 week before the first vaccine dose, an immunotherapy-mediated enhancement of the response cannot be excluded. To avoid ambiguities, all 3 patients were not considered for the statistical evaluation.

Amongst the 20 CPs, there were three patients that did not respond properly to the prime-boost vaccination. These were CP1 and CP8 from the cohort B; subgroup B.1 and CP18 from cohort C. All these patients did not generate S-specific IgGs, neutralising antibodies or virus-specific T cells. An additional two patients, CP6 (stage III melanoma treated with adjuvant anti-PD-1 plus bempegaldesleukin, cohort B, subgroup B.2) and CP14 (metastatic non-small cell lung cancer in partial response to chemotherapy plus anti-EGFR antibody, cohort C), responded weakly with borderline responses in all three assays. The remaining 15 CPs showed a vaccine response that was comparable to the healthy control group. Together this demonstrates an important immune response heterogeneity in solid CPs and argues for the need of a careful diagnostic follow-up.

Comparison of the 3 w and 9 w post-boost responses revealed a rather stable immunity with no significant differences within the cohorts (**Figures 1B, D, F**). Only CP2 (metastatic non-small cell lung cancer in complete response to anti-PD-L1 antibody; cohort B, subgroup B.1) had a drop in the S-specific IgG titre by two serum dilutions. As this was not accompanied by a loss of neutralising antibodies or Th1 responses, it was not considered clinically relevant. Patients CP3 (metastatic colorectal cancer in complete response to anti-PD-1 antibody; cohort B, subgroup B.1) and CP11 (metastatic non-small cell lung cancer in complete response to anti-PD-L1 antibody, cohort B, subgroup B.1) showed a reduction of their neutralising antibodies which was for CP11 accompanied by an over 2-fold reduction in the Th1 response. However, most notably was the strong increase of all three immune response measures for CP6 (stage III melanoma treated with adjuvant anti-PD-1 plus bempegaldesleukin, cohort B, subgroup B.2). This patient was a weak responder to the vaccine itself but then had a huge increase in virus-specific IgG titre, neutralising antibodies and Th1 response (**Figures 1B, D, F**; highlighted in red). Thus, the combination of a checkpoint inhibitor with long lasting interleukin 2 may trigger superior vaccine-induced immunity in an otherwise low responder.

## Discussion

Our study demonstrates a significant albeit variable immunogenicity of mRNA-based COVID-19 vaccines for patients with solid tumours under anti-PD1/PDL1 inhibitor immunotherapy or chemotherapy. Long lasting responders to cancer immunotherapy did not have higher responses to COVID vaccines, although there was a relevant case with an exaggerated response during cancer treatment with the combination of anti-PD-1 antibody plus bempegdesleukin. There was no relevant toxicity due to vaccination nor cancer treatment. These data are in line with previous findings (summarised in **Tables 2**, **3**) and enable altogether the following general and clinically relevant conclusions. (1) mRNA-based COVID-19 vaccines are highly immunogenic and safe even in immunocompromised solid tumour patients under various therapy regimens. (2) Roughly up to 30% of solid tumour patients may not generate a proper vaccine response and thus require additional boosters. (3) The biology of the cancer type (i.e. haematological tumour vs solid tumour) dictates vaccine efficacy. (4) The therapy regimen may affect immune response induction and duration of immune memory.

**Table 2:** Summary of COVID-19 vaccination trials in cancer patients under diverse therapies.

Author/ Country/ Reference	Total patients [n]		Type of cancer		Median age (range) [years]		Patient received vaccine [n]			Patients on active treatment [n]	Cancer treatment				
	CP	Control group	Patients with solid tumor [n]	Patients with hematological cancer [n]	CP	Control group	BNT162b2	mRNA-1273	Ad26.COV2.S		never treated	CHT and IMT	CHT	IMT	TT
(3)	44	0	0	44 (CCL)	71 (37–89)	n.a.	25	19	0	18	26	n.a.	n.a.	14	n.a.
(14)	816	274	816	0	62 (21-97)	47 (21-69)	CP: 786; Control: 251	0	0	738	n.r.	55	240	228	215
(16)	151	54	95	56	73.0 (64.5–79.5)	40·5 (31·3–50·0)	SC: 25; HC: 6; Control: 16	0	0	13	SC: 9/25; HC: 4/6	SC:1/25	SC: 4/25	SC:3/25	n.a.
(18)	232	261	232	0	68 (25-88)	64 (25-81)	CP: 218	0	0	232	0	n.a.	134	83	n.a.
(9)	167	52	0	167 (CLL)	71 (63-76)	68 (64-74)	219	0	0	75	58	n.a.	n.a.	n.a.	n.a.
(20)	36	72	26	10	82 (80-89)	n.r.	108	0	0	31	n.r.	11	10	2	n.a.
(21)	102	78	102	0	66 (56-72)	62 (49-70)	180	0	0	102	0	14	30	22	n.a.
(22)	92	36	0	MM: 42; MPM: 50	MM:73 (47–78); MPM:70 (28–80)	81 (79–87)	128	0	0	92	0	n.a.	n.a.	n.a.	n.a.
(23)	200	26	134	66	67 (27–90)	64 (37-82)	115	62	20	135	11	n.a.	112	54	n.a.
(24)	163	0	163	0	66 (27-89)	n.r.	163	0	0	163	0	0	122	15	26
(25)	171	2406	171	0	68 (58-73)	48 (36-56)	150	21	0	171	0	4	110*	23*	78*
(26)	176	25	49	91	SC: 66 (31-81); HC: 71 (47-97)	68 (28-90)	106	95	0	140	n.r.	n.r.	n.r.	n.r.	n.r.
(27)	60	0	60	0	66 (60-71)	n.r.	60	0	0	60	0	10	0	50	0
(28)	75	0	75	0	68 (61.5-73)	n.r.	75	0	0	75	0	20	0	66	4

Abbreviations: EC50, 50% effective concentration; CLL, chronic lymphocytic leukemia; CI,confidence interval; CP, Cancer patients; n, numbers; nAbs, neutralizing antibodies; MM, multiple myeloma; MPM, myeloproliferative malignancy; n.r., not reported; n.a., not applicable; Th1, T helper 1 cells; SC, solid cancer; HC, hematologic cancer; IQR,interquartile range; GMC, geometric mean concentration; CHT, chemotherapy; IMT, immunotherapy; TT, targeted therapy; anti-S, anti-spike protein; IFNγ,interferon-γ

*Total column values may not sum up to total as categories are not mutually exclusive (e.g. participants may have received CHT and TT)

**Table 3:** Summary of immune responses of COVID-19 vaccination trials in cancer patients under diverse therapies.

Author/ Country/ Reference	Positive Cutoff/ Unit			Number of individuals with vaccine-specific immune response											Patients’ Median Serum IgG Level (Post-Vaccinations)	Controls’ Median Serum IgG Level (Post-Vaccinations)
				anti-S IgG							nAbs		Th1 (IFNγ-ELISpot)			
	anti-S IgG	nAbs	Th1 cells	never treated, [n]/ total [n] (%)	CHT and IMT, [n]/ total [n]	CHT, [n]/ total [n] (%)	IMT, [n]/ total [n] (%)	TT, [n]/ total [n] (%)	CP, [n]/ total [n] (%)	Control, [n]/ total [n] (%)	CP, [n]/ total [n] (%)	Control, [n]/ total [n] (%)	all CP, [n]/ total [n] (%)	Control, [n]/ total [n] (%)		
(3)	IgG ≥15 AU/ml	n.a.	n.a.	17/18 (94%)	n.a.	n.a.	2/14 (14%)	n.a.	23/44 (52%)	n.a.	n.a.	n.a.	n.a.	n.a.	n.r.	n.r.
(14)	IgG ≥15 AU/ml	Co/S > 10	n.a.	n.r.	n.r.	230/261 (88.1%)	126/129 (97.7%)	195/201 (97%)	181/ 194 (93.3%)	204/204 (100%)	240/301 (79.7%)	131/175 (74.9%)	n.a.	n.a.	236.37 (13.77-4055.91)	262.98 (101.42-681.96)
(16)	≥ 70 EC_50_ dilution units	n.r.	≥7 cytokine secreting cells/10^6^	n.a.	n.a.	n.a.	n.a.	n.a.	SC: 18/19 (95%); HC: 3/5 (60%)	12/12 (100%)	n.a.	n.a.	SC: 14/16 (87.5%) ; HC: 3/4 (75%)	3/3 (100%)	n.r.	n.r.
(18)	IgG ≥15 AU/ml	n.a.	n.a.	n.a.	n.a.	102/ 125 (82%)	71/79 (89%)	n.a.	187/218 (86%)	n.r.	n.a.	n.a.	n.a.	n.a.	n.r.	n.r.
(19)	IgG ≥0.8 U/ml	n.a.	n.a.	32/58 (55%)	n.a.	n.a.	n.a.	n.a.	66/167 (39.5%)	52/52 (100%)	n.a.	n.a.	n.a.	n.a.	155 (IQR, 7.6-490.3) U/mL	1084 (IQR, 128.9–1879) U/ml
(20)	IgG ≥50 AU/ml	n.a.	n.a.	n.a.	n.r.	n.r.	n.r.	n.a.	SC: 25/26 (96%); HC: 4/10 (40%)	12/12 (100%)	n.a.	n.a.	n.a.	n.a.	2396.10 (range 0–32,763) AU/ml	8737.49 (range 398.90–976,280) AU/ml
(21)	IgG ≥50 AU/ml	n.a.	n.a.	n.a.	n.r.; Median IgG titer: 310 AU/ml	n.r.; Median IgG titer: 1363 AU/ml	n.r.; Median IgG titer: 3020 AU/ml	n.a.	92/102 (90%)	78/78 (100%)	n.a.	n.a.	n.a.	n.a.	1931 (IQR, 509–4386) AU/ml	7160 (IQR, 3129–11,241) AU/ml
(22)	IgG ≥15 AU/ml	n.a.	n.a.	n.a.	n.a.	n.a.	n.a.	n.a.	MM:33/42 (79%); MPM:44/50 (88%)	36/36 (100%)	n.a.	n.a.	n.a.	n.a.	GMC (CI): MM 106.7 (62.3–179.7) AU/ml; MPM 172.9 (106.5–257.0) AU/ml	GMC (CI): 353.3 (255.6–470.0) AU/ml
(23)	IgG ≥50 AU/ml	n.a.	n.a.	n.a.	n.a.	103/112 (92%)	46/54 (85%)	n.a.	SC: 131/134 (98%); HC: 56/66 (85%)	26/26 (100%)	n.a.	n.a.	n.a.	n.a.	SC: 7858 AU/ml; HC:2528 AU/ml	Higher than 15,000 AU/ml
(24)	IgG ≥1000 AU/ml	n.a.	n.a.	n.a.	n.a.	66/106 (62 %)*	12/14 (86%)*	14/22 (64%)*	92/142 (65%)*	n.a.	n.a.	n.a.	n.a.	n.a.	1996.3 (IQR 498.2-5575.3) AU/ml	n.a.
(25)	IgG ≥33.8 AU/mL	nAb ≥20 %	n.a.	n.a.	n.r.	102/110 (92.7%)	22/23 (95.7%)	73/78 (93.6%)	161/171 (94.2%)	2402/2406 (99.8%)	122/161 (76,2%)	n.a.	n.a.	n.a.	n.a.	n.a.
(26)	IgG ≥500 U/mL	n.a.	n.a.	n.a.	n.a.	n.a.	n.a.	n.a.	SC: 27/49 (55.1%); HC: 7/91(7.7%)	59/61 (96.7%)	n.a.	n.a.	n.a.	n.a.	n.a.	n.a.
(27)	IgG ≥ 15 AU/mL	nAb ≥ 1:10	≥ 10 net spot/million PBMCs	n.a.	n.a.	n.a.	72/75 (95%)	n.a.	72/75 (95%)	n.a.	n.r.; 1:80 (IQR 1:20-1:160)	n.a.	n.r; Median: 125 (IQR 52.5-345) IFN-g SFU/million PBMCs	n.a.	n.a.	n.a.
(28)	IgG ≥ 33.8 BAU/mL	nAb ≥ 1:10	≥ 10 net spot/million PBMCs	n.a.	n.r.; Median IgG titer: 80.6 BAU/ml	n.a.	n.r.; Median IgG titer: 196.3 BAU/ml	n.a.	n.r.; Median: 205.2 (IQR 73.2-654.6)	n.a.	n.r.; Median: 1:20 (IQR 1:10-1:40)	n.a.	n.r.; Median 50 (IQR 20-118.8) IFN-g-producing cells/million PBMCs	n.a.	n.a.	n.a.

Abbreviations: EC50, 50% effective concentration; CLL, chronic lymphocytic leukemia; CI,confidence interval; CP, Cancer patients; n, numbers; nAbs, neutralizing antibodies; MM, multiple myeloma; MPM, myeloproliferative malignancy; n.r., not reported; n.a., not applicable; Th1, T helper 1 cells; SC, solid cancer; HC, hematologic cancer; IQR,interquartile range; GMC, geometric mean concentration; CHT, chemotherapy; IMT, immunotherapy; TT, targeted therapy; anti-S, anti-spike protein; IFNγ,interferon-γ

* Values obtained one month after the 3rd vaccination were: for patients under CHT 73% (22/30), for patients with TT 83% (5/6) and for IMT no patients were analyzed. 75% (27/36) of the CP seroconverted.

Our study included only 20 solid CPs under diverse therapies and thus is limited in its informative value. We therefore reviewed previously published work and included their results, as summarised in **Tables 2**, **3**, to obtain the mentioned conclusions. In particular, the work of Thakkar et al., 2021 demonstrates the significant differences of the cancer type and therapy regimen on vaccination efficacy. Given that vaccines rely on the coordinated functioning of antigen-presenting cells and B and T lymphocytes, it is not surprising that haematological tumours and their therapies which affect these cell types, impede vaccine responses the most (23). Furthermore, since the induction of a vaccine response requires lymphocyte proliferation and differentiation, chemotherapies that target dividing cells are expected to influence this process negatively and thus reduce vaccine efficacies. Indeed, this is observed albeit the differences, for example, in SARS-CoV-2-S-specific antibody titres for CPs with or without chemotherapy were not statistically significant (23). However, the response level between the patients was very divergent and it may well be that the timing between cytotoxic drug intake and the vaccine schedule might have an important influence (29). Detailed studies on this are still lacking and clearly require further attention.

The three vaccine non-responders and the two low-responders represent together 25% of our patient group. They were low in both humoral and cellular immune responses. Importantly, one of them received bempegdesleukin that significantly boosted both types of responses suggesting that this low-responsive subgroup of patients has no inherent defect to respond and thus may benefit from booster vaccinations or combinations with other immune modulating drugs (29). This should particularly be true for vaccines, like the ones used here, that induce antibodies and cellular immunity because both antiviral effector mechanisms seem to cooperate in antiviral defence in a multiplicative, synergistic way (30).

Immunotherapy with checkpoint inhibitors is a common treatment option for patients with solid tumours. Respective therapeutic antibodies disrupt inhibitory signaling pathways in lymphocytes and thereby augment cellular immune responses. In the context of antiviral vaccines, this might have the immunological benefit of generating increased immune responses and thus better host protection against subsequent virus infections. However, our study revealed no significant differences in the vaccine responses between the immune cancer responders (cohort B) and the CPs not-treated with immunotherapy (cohort C), and both cohorts contained individuals that lacked an efficient response. Furthermore, there were no additional side effects compared to the healthy control group suggesting that the mRNA-based vaccine regimen is safe and well tolerated in solid CPs independent on the type of therapy, including those patients with excellent response to cancer immunotherapy. These findings are in line with those of others (2, 14, 23, 27, 29, 31) and support the current recommendations to vaccinate CPs against COVID-19 independent of their therapy regimens (14).

In conclusion, our results here and the very recent findings of others consistently support COVID-19 vaccination of tumour patients to reduce their enhanced mortality risks upon SARS-CoV-2 infection. The existence of a significant portion of low-vaccine responders argues for diagnostic follow-up and booster vaccinations or novel combinations. Further studies that evaluate and optimise the timing between cancer therapies and vaccination schedules are warranted.

## Data Availability Statement

The raw data supporting the conclusions of this article will be made available by the authors, without undue reservation.

## Ethics Statement

The studies involving human participants were reviewed and approved by “Grupo Hospitalario Quirónsalud-Catalunya”. The patients/participants provided their written informed consent to participate in this study. Written informed consent was obtained from the individual(s) for the publication of any potentially identifiable images or data included in this article.

## Author Contributions

Concept and funding acquisition: MG-C and AM. Experimental design: MS-O, MG-C and AM. Experiment performance: MS-O, DDJW and NA. Patient recruitment and handling: MG-C, RR, AAg and RG-F. Sample collection: AAg, CM, FGC, ST, EBS, LRS, AAr, CE, BG, JV, RR and RG-F. Figures and tables: MG-C, MS-O, DDJW and NA. Manuscript drafting: MS-O, DDJW and NA with corrections from AM, RR and MG-C. All authors contributed to the article and approved the submitted version.

## Funding

The authors are supported by Spanish Melanoma Group Grant (III Beca GEM para Grupos Emergentes), the Spanish Ministry of Science and Innovation grant no. PID2019-106323RB-I00 AEI//10.13039/501100011033, the “Unidad de Excelencia María de Maeztu”, funded by the MCIN and the AEI (DOI: 10.13039/501100011033); Ref: CEX2018-000792-M.

## Conflict of Interest

The authors declare that the research was conducted in the absence of any commercial or financial relationships that could be construed as a potential conflict of interest.

## Publisher’s Note

All claims expressed in this article are solely those of the authors and do not necessarily represent those of their affiliated organizations, or those of the publisher, the editors and the reviewers. Any product that may be evaluated in this article, or claim that may be made by its manufacturer, is not guaranteed or endorsed by the publisher.

## Supplementary Material

Supplementary Figure 1 Percentages of responders from cohorts A, B.1, B.2 and C upon COVID-19 vaccination. The percentage of responders from Cohorts A (healthy donors), B.1 (CP under IMT, long lasting responders), B.2 (CP under IMT, adjuvant therapy) and C (CP not treated with IMT, no-IMT) in ELISA **(A)**, NeutraLISA **(B)** and ELISPOTs **(C)** after two doses of mRNA-based COVID-19 vaccines (3 w post boost time-point). Cohort A is represented by black bars, cohort B.1 by light blue bars, cohort B.2 by dark blue bars and cohort C by red bars. The ratio of responders to group size is given above each individual bar.

Supplementary Figure 2 SARS-CoV-2-specific immune responses from cohort B subgroups B.1 and B.2 after COVID-19 vaccination. Spike-specific IgG titers **(A)**, nAb titers **(B)** and Spike-specific T cell responses **(C)** in CPs under immunotherapy (anti-PD1/PD-L1) from subgroup B.1 (long lasting responders) and subgroup B.2 (adjuvant therapy) after two doses of mRNA-based COVID-19 vaccines. Dotted lines indicate positive thresholds for Spike-specific IgG and T cells. In case of nAbs **(C, D)**, the dotted line determines the thresholds for negative, intermediate, and positive results according to manufacturer’s instructions. Bars represent medians.
