# Supplementary figures and images for: Brief Research Report: Anti-SARS-CoV-2 Immunity in Long Lasting Responders to Cancer Immunotherapy Through mRNA-Based COVID-19 Vaccination
Source: Front Immunol. 2022 Jul 5;13:908108. doi: 10.3389/fimmu.2022.908108 (PMC9330498; doi:10.3389/fimmu.2022.908108)

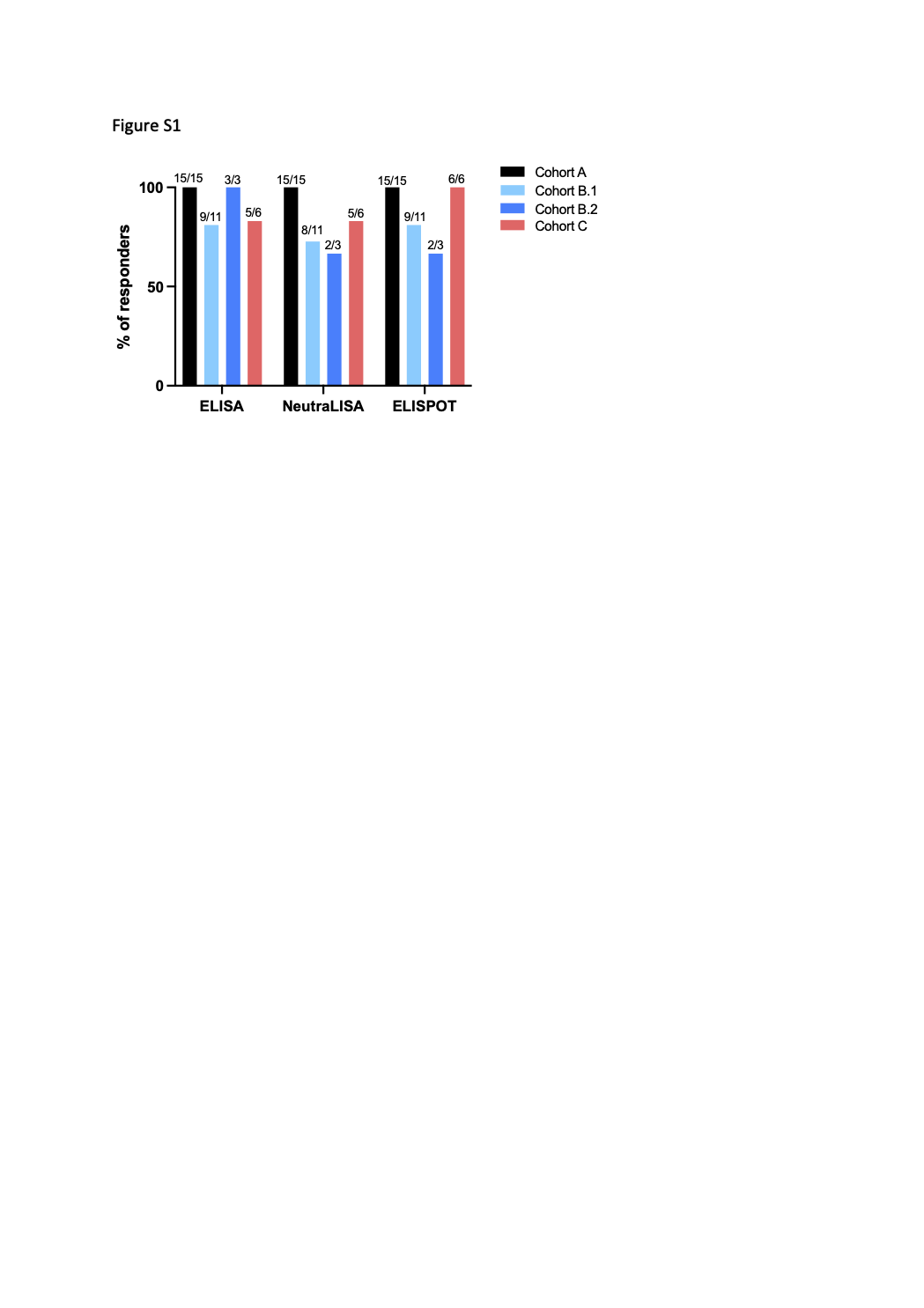

Supplement: Supplementary Figure 1 — Percentages of responders from cohorts A, B.1, B.2 and C upon COVID-19 vaccination. The percentage of responders from Cohorts A (healthy donors), B.1 (CP under IMT, long lasting responders), B.2 (CP under IMT, adjuvant therapy) and C (CP not treated with IMT, no-IMT) in ELISA (A), NeutraLISA (B) and ELISPOTs (C) after two doses of mRNA-based COVID-19 vaccines (3 w post boost time-point). Cohort A is represented by black bars, cohort B.1 by light blue bars, cohort B.2 by dark blue bars and cohort C by red bars. The ratio of responders to group size is given above each individual bar. [file Image_1.tiff]

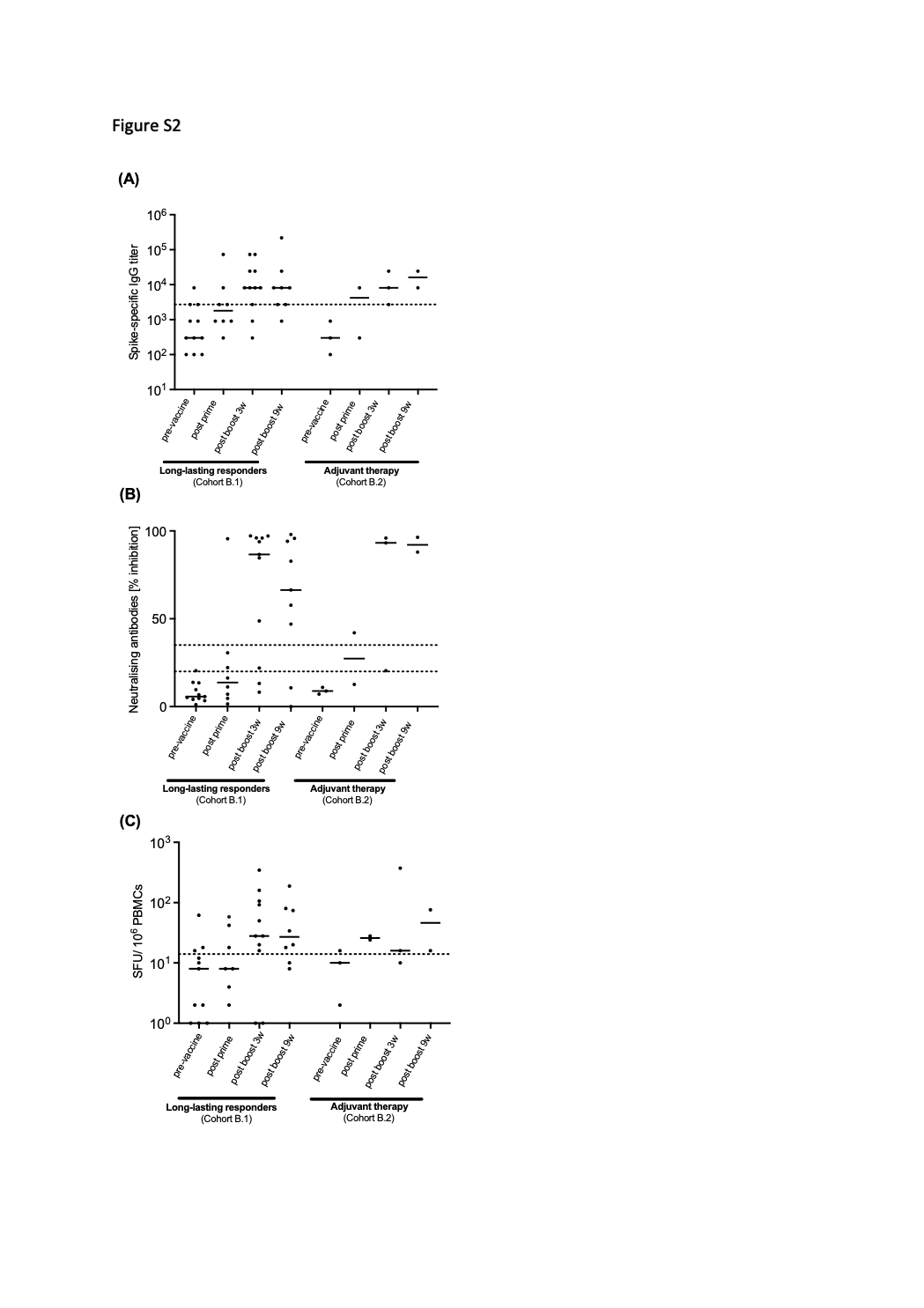

Supplement: Supplementary Figure 2 — SARS-CoV-2-specific immune responses from cohort B subgroups B.1 and B.2 after COVID-19 vaccination. Spike-specific IgG titers (A), nAb titers (B) and Spike-specific T cell responses (C) in CPs under immunotherapy (anti-PD1/PD-L1) from subgroup B.1 (long lasting responders) and subgroup B.2 (adjuvant therapy) after two doses of mRNA-based COVID-19 vaccines. Dotted lines indicate positive thresholds for Spike-specific IgG and T cells. In case of nAbs (C, D), the dotted line determines the thresholds for negative, intermediate, and positive results according to manufacturer’s instructions. Bars represent medians. [file Image_2.tiff]
